# Artificial Dentures in Their Relation to the Speaking or the Singing Voice

**Published:** 1898-12

**Authors:** Dwight L. Hubbard

**Affiliations:** New York


### ARTIFICIAL DENTURES IN THEIR RELATION TO THE SPEAKING OR THE SINGING VOICE.[1]

BY DWIGHT L. HUBBARD, M.D., NEW YORK.

To the crowning glory of the handiwork of the Creator is given that which none of the lower animals possesses,—viz., a power to express thought and a soul which finds a medium in articulate sounds. The Giver of this faculty gave to other creatures the same power in less degree; to some the intelligible manifestation of fear, pain, anger, and other emotions; to each according to its necessity. But to the greatest of His creatures He gave not only the power of expressing emotion by sign or articulate sound, but the power of original and logical thought in speech and song. In order, therefore, to treat this subject intelligently it may be well to indulge ourselves in a short consideration of comparative anatomy. Every

---

[1] Read before the Northeastern Dental Association, held at Hartford, Conn., October 19 and 20, 1898.

bird, every animal, is endowed with a voice according to its need. To some it is given for self-protection, for the acquirement of food, for the warning of impending danger; to others, of a higher type, for pleasure, for companionship, and for ministration to man and the glory of the Creator. Each and every one according to the necessity of its environment.

The beautiful song of the nightingale has its use in innocent glorification as well as in a contribution to poetic æstheticism. Let us look at the subject from a mechanical stand-point. It is very pleasant to dwell upon higher and æsthetic things, but serious business calls us from the realms of the beautiful, and reminds us that man lives by bread, but does not live by bread alone.

The voice is the highest type of mechanism in acoustics.

The squirrel, chipmonk, and others of the same family have voices sharp and shrill, but there is no resonance. They have flattened heads and very few resonance-chambers. The jackass (pardon me for so commonplace a reference) has a resonance capable of disturbing the peace of solitary dwelling-places or the minds of adjoining neighbors, either of his own kind or of a higher order. He has a large head when compared with his body. He has wide and large nasal cavities. He has a "large" voice with great carrying quality. The crow has a voice in quality and timbre far from musical, and represents a type of the "throaty" voice. This bird has a flattened head and no resonance-chambers. The canary has a voice of great sweetness and resonance. He has a high forehead and a comparatively enormous frontal sinus. The parrot has a voice of penetrating and carrying quality, and in accordance it will be observed that his beak is altogether in harmony with his vocal resonance, giving a remarkable variation almost human. Observe the resonance-chambers of the owl, and go to his haunts and be charmed by his music. The high and wide resonance-chambers of the lark have justly earned for him poetic tributes. H. H. Boyesen thus writes:

"The bird is to him but a winged symbol of divine unrest. The lark has caught the key-note of the poet's song and loses its earthly individuality."

Take exception, if you will, in the case of the bullfrog, but look at the air-pouch in his white throat. It is out of all proportion to the size of his head. His deep diapason may put to shame the attempts of a contrabasso.

So in man, resonance-chambers are a necessity to good voice-production. The high forehead with its frontal sinus, the porous bones of the upper face, honey-combed with cells, the thirty-six square inches of nasal space, the vaulted dome of the naso-pharynx, all lend resonance to the thought medium. The anatomical construction of the face and head should not be forgotten.

It should be borne in mind that hyperplastic inflammation of the lymphatic glands of the pharyngeal, laryngeal, and cervical regions is liable to take place as the result of disturbances in the teeth and also in contiguous structures. Neither should the extension of possible inflammation to the sinuses be neglected. The relationship of these cavities to the parts named is very intimate. We have only a thin mucous membrane covering the palatal foramen, and a direct communication with the antrum of Highmore with the nasal cavity. These are in intimate association with the oral cavity. So with the other sinuses, the frontal connecting with the nasal cavity by means of the infundibulum, as pointed out particularly by Dr. Fillebrown; the ethmoidal cells in direct communication with the nasal chambers and the sphenoidal sinus, very liable to disease from extending catarrhal affections; all of which may be caused by tooth-disturbances. I speak of these only because they have a direct bearing upon voice-production, and to particularly fix a partial responsibility upon those whose business it is to look after primary causes.

In order to prove that vibration of the air in the nasal cavity takes place during phonation, place a vulcanized rubber splint of small size in the nostril, and upon phonation the singing quality and the vibration will be plainly felt. To demonstrate that a part of this vibration is communicated through the palatal bones and foramen, hold against the hard palate a sufficiently large piece of wax, and the diminution in the nasal and head vibration will be very noticeable. I wish merely to show that artificial appliances in the roof of the mouth are hinderances to proper phonation, and that some attention should be given to the avoidance of such possibilities. Aside from the soul of music in the singer, everything depends upon the tone-placement. No obstruction, no matter how slight, should be placed in the way, whether it be an obturator, a plate, or a simple filling, unless necessary. When necessary, due attention should be given to the best tone-producer, whether it be required for grand opera, the rostrum, or common conversation. As the crowning work man stands unique in that he must *learn* the art of speech and song; but is not this a tribute to his greater intelligence? How much more, then, should he seek to acquire perfectness in this one of the highest gifts.

What relation have artificial dentures to these cavities? When a violin string becomes "fuzzy" the tone is muffled. It no longer gives forth a clear note. Puncture a brass instrument and solder it with lead, and its clarion notes lose their clearness. Place an iron weight upon the sounding-board of a piano, and it ceases to give forth the harmonies of the mind of its master. The human vocal instrument can no more tolerate these obstructions than can those used in the illustrations.

I will not refer to pathological conditions, nor enter into a discussion of them, except as they place hinderances in the way of clear voice-production and the necessity for their recognition in the fitting of dentures and of dental operations in general. *Apropos* to what has already been said, let us take as our first principle that the resonance-chambers must be free from all obstruction. By resonance-chambers I refer to all the cavities of the face, including the nasal and naso-pharyngeal spaces and the oral cavity. The last is not least in importance.

Teachers of elocution and singing-masters lay particular stress upon "tone-placement." By this is meant such a focussing of sound-waves that the air in the spaces referred to may be made to vibrate the most readily. This gives carrying power to the voice. In other words, the air of an auditorium can be made to vibrate only when the vibration is started in the vocal mechanism. The vocal cords simply act as the initial mechanism of the tone, the resonance-chambers being really the tone-producers. Vocal cords alone are nothing more than a violin-string without the body of the violin. The choicest place for focussing sound-waves seems to be in the palatal arch, its exact location varying in different individuals according to the shape of the arch and the size and relative positions of the different sinuses. The palatal arch is a most interesting locality to the dentist. At this point a good or bad denture may retain, make, or mar the tone quality and timbre. It is a self-evident fact, then, that perfect fitting and perfect finish are of great importance. Second, it is of no less importance that we should consider the quality of material used as also the material itself. It follows that as little interference as possible to the conduction of sound should be placed in the way. The focus of vibration at the point mentioned is largely instrumental in communicating sympathetic vibration to the nasal cavity. For perfect fitting you, of the dental profession, must be depended upon, for with reference to this I cannot speak with authority. As to the material to be used I have but little to say, but that little will be from experience. We know that vulcanized rubber is almost as poor a conductor of sound as it is of electricity. We know that porosity hinders the conduction of sound and that density increases it. The metals whose chief characteristic is that of fibrous density are the best conductors. But it is sometimes easy to do our work too well. Suppose we could use a plate of steel. We would thus overdo the work and get too much of the metallic conduction out of all proportion to the capacity of the resonance-chambers. Then we must look for a happy medium which will be proportionate in their capacity. The material must be neither too soft nor too dense. Its density must be varied according to the individual case, other necessary things being also considered as a matter of course. You have reached such a stage of perfection in the management of gold that it is now a comparatively easy thing to obtain the proper and desired density appropriate to each case. But with any metal as a "*back*" it seems to me that a modifier should be used in combination with it. To sum up this part of my subject, it would seem that a vulcanite plate of perfect finish, as dense as possible, backed with gold of proper density would be an ideal plate.

The same principles should be observed with regard to artificial teeth and fillings. It may be asked if the teeth have anything to do with resonance. In answer, and to prove that they most decidedly do, that the so-called "throaty" voice produces no vibration either in the resonance-chambers or in the teeth, let us observe how the bones of the face must take part in the vibration of properly produced tones, both in speaking and singing. Produce a "throaty" tone, coming only from the larynx, place the teeth of the lower and upper jaws very gently in apposition, and no vibration is felt. Now produce a tone in which all the resonance-chambers are used with the teeth in apposition, and the vibration is so distinctly felt as to become exceedingly uncomfortable. *Apropos* also to this, I would establish the principle that speakers and singers should have good teeth, whether they be natural or artificial; also that the occupation of your patient should be considered. In this regard, advancement in mechanical dentistry has happily furnished good material especially applicable to the cases here considered. How often do we find that nature is so perfectly balanced that discoveries and appliances often cover a wider range of necessities than that for which they were first made.

The above are only hints. I am here as much for the social pleasure of being with you as to try to add to the scientific interests of your meeting. I am reminded that the possibilities for expansion in the application of dental science are great. As dentistry is rapidly broadening into surgical fields, it may not be assumption to think how it may come to pass that you may some day find it necessary to become elocutionists and singing-masters. "Music hath charms to soothe even the savage breast."

But, seriously, is it not time that we realized the fact that a study of any part of the human body involves necessarily a study of the whole?

May I digress for a moment, and call to your attention that the main pages in our dental journals are devoted largely to some reflections upon some imaginary or real violation of suggested therapeutics; to some proposed theory as to the management of a society, or to ethics in some remote corner of the world? Please pardon me for the suggestion, but would it not be better if we could broadly and optimistically pour the milk of human kindness all over this land, and at the same time think of and act for the good of our fellow-men in the things which will make him physically happier? Surely our efforts would be well repaid in the certainty that we have made him better; the result of which will be the working out of his own individuality, that high ideal for which he was created. Let us live the broader, the higher life.

I have, I know, really said nothing, but if, coming from one who is in sympathy with you as brethren, I have added even the smallest quota of enjoyment or profit, I shall feel that I might have come to a worse place. I shall never hope to go to a better one until the summons comes to go one step higher into the land where dental pathology is unknown and where oral surgery is not required.

